# Protein–β-Glucan Nanoparticle-Enriched Peach Juice: In Vitro Bioaccessibility, Metabolic Enzyme Inhibition, and Sensory Evaluation

**DOI:** 10.3390/foods15162818

**Published:** 2026-08-12

**Authors:** Monika Stojanova, Marina S. T. Stojanova, Dragutin A. Djukic, Olga Popovska, Arita Sabriu Haxhijaha, Yalcin Kaya

**Affiliations:** 1 Faculty of Technological Sciences, Mother Teresa University, 1000 Skopje, North Macedonia; olga.popovska@unt.edu.mk (O.P.); arita.haxhijaha@unt.edu.mk (A.S.H.); 2 Faculty of Agricultural Sciences and Food, University of Ss. Cyril and Methodius, 1000 Skopje, North Macedonia; 3 Faculty of Agronomy, University of Kragujevac, 32000 Cacak, Serbia; open.science@yahoo.com; 4 Engineering Faculty, Trakya University, 22030 Edirne, Türkiye; yalcinkaya@trakya.edu.tr

**Keywords:** peach juice, protein–β-glucan nanoparticles, functional beverages, gastrointestinal digestion, bioaccessibility

## Abstract

This study introduces a novel food-grade protein–β-glucan nanoparticle system based on *Fusarium venenatum* protein isolate and *Fuscoporia torulosa* β-glucans, designed as a multifunctional ingredient for improving the physicochemical stability, bioaccessibility, and functional performance of peach juice beverages. Therefore, this study aimed to develop and evaluate a functional peach juice enriched with protein–β-glucan nanoparticles and to investigate their effects on physicochemical stability, gastrointestinal behavior, metabolic enzyme inhibition, antioxidant retention, and sensory acceptance. Protein–β-glucan nanoparticles produced from *Fusarium venenatum* protein isolate and *Fuscoporia torulosa* β-glucans were incorporated into peach juice formulations (V1–V4) and characterized using dynamic light scattering, zeta potential analysis, transmission electron microscopy (TEM), and Fourier-transform infrared spectroscopy (FTIR). In vitro gastrointestinal digestion was performed to assess β-glucan bioaccessibility, protein digestibility, antioxidant activity, and release kinetics, while α-amylase and α-glucosidase inhibition assays, sensory evaluation, digital sensory analysis, principal component analysis (PCA), and exploratory multivariate analysis were used to evaluate relationships among formulation characteristics and functional performance. The nanoparticles formed stable colloidal systems with mean particle sizes ranging from 176 to 229 nm and maintained structural integrity during 28 days of refrigerated storage. Nanoparticle-enriched formulations exhibited higher β-glucan bioaccessibility, improved antioxidant retention, and stronger α-amylase and α-glucosidase inhibitory activities, with the greatest effects observed in V4. Protein digestibility remained high across all formulations, while sensory evaluation revealed improved overall liking and purchase intention. PCA indicated that nanoparticle concentration was the main factor differentiating the formulations based on their physicochemical and functional characteristics. Overall, protein–β-glucan nanoparticles enhanced the physicochemical, functional, and sensory properties of peach juice, supporting their application as multifunctional ingredients for functional beverage development.

## 1. Introduction

Functional foods have attracted increasing scientific and industrial interest due to their potential to provide health benefits beyond basic nutrition through the incorporation of bioactive compounds [1,2,3,4].

Among bioactive compounds, β-glucans have received considerable attention due to their diverse physiological properties. These naturally occurring polysaccharides, present in mushrooms, cereals, and other biological sources, have been associated with immunomodulatory activity, cholesterol-lowering effects, and improved glycemic regulation [2]. However, their application in functional food systems may be limited by structural instability, interactions with food matrices, and changes during processing and storage, which can influence their bioavailability and functional performance [5,6,7]. These limitations are strongly influenced by the physicochemical characteristics of β-glucans, including molecular weight, degree of branching, solubility, and hydration behavior. High-molecular-weight β-glucans may provide desirable viscosity and gel-forming properties; however, excessive viscosity can negatively affect dispersion, mouthfeel, and processability in beverage systems. In addition, β-glucans can undergo structural changes during thermal processing and storage, while interactions with proteins, polyphenols, and other matrix components may alter their solubility, accessibility, and functional performance. Therefore, controlling β-glucan organization within suitable delivery systems is essential to improve its stability and applicability in complex food matrices. Therefore, the development of suitable systems capable of improving the stability and functionality of β-glucans represents an important challenge in functional food design.

Nanotechnology-based approaches have recently gained attention as potential strategies for improving the dispersion, stability, and bioaccessibility of bioactive compounds. Reduction in particle size to the nanoscale can increase the surface-area-to-volume ratio and enhance interactions with food components and digestive systems [8]. In beverage applications, nanoparticle-based systems may provide opportunities for incorporating functional ingredients while maintaining desirable physicochemical and sensory properties. However, information regarding the application of β-glucan-based nanoparticles, particularly protein–β-glucan systems, in fruit juice matrices remains limited. The interactions between these systems and digestive processes, including bioactive release, enzyme inhibition, antioxidant retention, and sensory performance, require further investigation [9].

At the same time, increasing consumer preference for clean-label products has encouraged the exploration of natural strategies for improving the functionality and quality of processed fruit beverages. The development of biopolymer-based systems using naturally derived components represents a promising approach for enhancing the functional properties of beverages while reducing reliance on conventional synthetic ingredients [9,10].

Previous studies investigating the incorporation of dietary fibers and other bioactive compounds into fruit juices have demonstrated improvements in antioxidant capacity, inhibitory activity against digestive enzymes, and sensory characteristics [11]. Nevertheless, many studies have focused on individual functional properties rather than evaluating the relationship between bioaccessibility, gastrointestinal release behavior, digestive responses, antioxidant stability, and consumer acceptance within a single beverage system. Consequently, the potential of advanced β-glucan-based delivery systems to simultaneously influence these properties remains insufficiently explored [12,13,14].

A comprehensive evaluation of functional beverages requires consideration of both biological functionality and product quality. The gastrointestinal bioaccessibility of β-glucans influences their potential physiological effects after consumption [13]. Similarly, α-amylase and α-glucosidase inhibition represents an important mechanism associated with the regulation of postprandial glucose responses. In addition, antioxidant retention during digestion and sensory acceptance are essential parameters determining the practical applicability of novel beverage formulations [14,15].

Considering these aspects, further research is needed to clarify the role of protein–β-glucan nanoparticle systems in complex food matrices such as fruit juices. Limited information is currently available regarding the application of microbial-derived protein–β-glucan nanoparticles, particularly systems based on Fusarium venenatum protein isolate and Fuscoporia torulosa β-glucans, for improving the physicochemical, functional, and sensory properties of fruit beverages.

Therefore, the present study aimed to develop a functional peach juice enriched with protein–β-glucan nanoparticles and to evaluate its physicochemical, functional, metabolic, and sensory characteristics. Specifically, the study investigated β-glucan bioaccessibility and release kinetics during simulated gastrointestinal digestion, protein digestibility, antioxidant retention, and inhibitory activity against α-amylase and α-glucosidase. Furthermore, consumer acceptance was assessed through hedonic evaluation and digital facial expression analysis. Finally, exploratory multivariate analysis was applied to investigate relationships among nanoparticle concentration, storage stability, bioactive release, functional performance, and sensory outcomes. This approach provides further insights into the potential application of microbial protein–β-glucan nanoparticles as naturally derived multifunctional ingredients for functional beverage development.

## 2. Materials and Methods

### 2.1. Microorganism and Culture Conditions

*Fusarium venenatum* was selected as the microbial protein source based on its high protein productivity and well-established techno-functional properties for biomass-based applications [16]. Cultures were maintained on malt extract agar (MEA) at 4 °C and subcultured every four weeks. Spore suspensions were prepared from 7-day-old MEA cultures using sterile distilled water containing 0.01% (*v*/*v*) Tween 80 to facilitate spore dispersion. Spore concentration was determined using a hemocytometer and adjusted to 1 × 10^6^ spores/mL for inoculation. All experiments were performed in triplicate under controlled laboratory conditions.

#### 2.1.1. Preparation of Solid Medium for Surface Fermentation

A solid medium containing starch (20 g/L), peptone (5 g/L), MgSO_4_·7H_2_O (1 g/L), and K_2_HPO_4_ (0.5 g/L) was prepared, adjusted to pH 6.0, and sterilized at 121 °C for 20 min. After cooling, the medium was aseptically distributed into shallow stainless-steel trays (2–3 cm depth), inoculated with *Fusarium venenatum* spore suspension (1 × 10^6^ spores/mL), and incubated under static conditions at 28 °C and ≥85% relative humidity for 7 days. Surface fermentation was performed under aseptic conditions in triplicate following a modified method of Reihani and Khosravi-Darani [17].

#### 2.1.2. Harvesting and Lyophilization

After 7 days, mycelial mats were aseptically harvested, washed three times with phosphate buffer (pH 7.0), and frozen at −80 °C for at least 12 h. Samples were freeze-dried at −50 °C under vacuum (<0.1 mbar) for 48 h. The resulting biomass was stored in airtight containers at −20 °C until nanoparticle synthesis, following Stojanova [18].

#### 2.1.3. Preparation of *Fusarium venenatum* Protein Isolate

Freeze-dried *Fusarium venenatum* biomass was milled and suspended in distilled water (1:10, *w*/*v*). The suspension was adjusted to pH 10.0 with 1 M NaOH and stirred for 2 h at room temperature, followed by centrifugation at 10,000× *g* for 20 min. The protein-rich supernatant was collected, adjusted to pH 4.5 with 1 M HCl to precipitate proteins, and centrifuged at 10,000× *g* for 20 min. The precipitated proteins were washed twice with distilled water, neutralized to pH 7.0, freeze-dried, and stored at −20 °C until nanoparticle preparation. The extraction procedure was adapted from previously reported methods for fungal protein isolation [19].

### 2.2. Preparation and Lyophilization of Fuscoporia torulosa Extract

Dried fruiting bodies of *Fuscoporia torulosa* were finely ground and extracted with distilled water at 80 °C for 2 h under continuous stirring. The extract contained approximately 32.9% β-glucan on a dry matter basis and was used as a β-glucan-enriched fraction. Insoluble residues were removed by centrifugation at 5000× *g* for 15 min, and the supernatant was frozen at −80 °C and lyophilized at −50 °C under vacuum (<0.1 mbar) for 48 h. The resulting β-glucan-enriched powder was stored in airtight containers at −20 °C until nanoparticle synthesis [20,21].

### 2.3. Synthesis of Protein-Based Nanoparticles

Lyophilized *Fusarium venenatum* protein isolate (3%, *w*/*v*) was dispersed in distilled water and mixed with lyophilized *Fuscoporia torulosa* extract at a protein-to-extract ratio of 10:1 (*w*/*w*). This ratio was selected based on preliminary optimization experiments evaluating nanoparticle formation and dispersion characteristics. The mixture was sonicated using a probe-type ultrasonic processor (VCX 130, Sonics & Materials Inc., Newtown, CT, USA) at 20 kHz and 50 W for 5 min in pulse mode (5 s on/5 s off) while maintained in an ice bath.

The suspension was subsequently heated at 70 °C for 10 min and cooled to room temperature (25 °C). The pH was adjusted to 6.8 using 0.1 M NaOH or HCl. The resulting dispersion was frozen and lyophilized at −50 °C under vacuum conditions (<0.1 mbar) for 48 h. The dried protein–β-glucan nanoparticles were stored in airtight containers at −20 °C until further use.

### 2.4. Application of Protein–β-Glucan Nanoparticles in Peach Juice

#### 2.4.1. Peach Juice Preparation and Experimental Design

Fresh peach juice was prepared from fully ripe fruits, filtered through a 200 μm mesh, and used immediately after preparation. Four formulations were evaluated: V1—commercial peach juice with additives (control), V2—freshly prepared juice without additives (negative control), V3—juice supplemented with 0.1% (*w*/*v*) protein–β-glucan nanoparticles, and V4—juice supplemented with 0.3% (*w*/*v*) protein–β-glucan nanoparticles. Nanoparticles were dispersed in juice under gentle stirring (300 rpm, 10 min) at room temperature. No thermal processing or synthetic additives were applied after incorporation. Formulations V1 and V2 served as control samples without added protein–β-glucan nanoparticles and were therefore not subjected to nanoparticle-specific characterization techniques, including DLS, zeta potential analysis, TEM, and FTIR. These formulations were evaluated in subsequent functional, digestion, and sensory analyses to compare the effects of nanoparticle incorporation. To minimize the risk of microbial contamination during sample preparation and storage, all glassware, containers, and mixing utensils were thoroughly cleaned and sterilized prior to use. Juice preparation and nanoparticle incorporation were carried out under aseptic laboratory conditions, and the formulations were immediately transferred into sterile, airtight containers and stored at 4 °C. Samples were handled only during scheduled analyses to minimize external contamination throughout the experimental period.

#### 2.4.2. Incorporation of Nanoparticles

Protein–β-glucan nanoparticles were added to peach juice at concentrations of 0.1% and 0.3% (*w*/*v*) and homogenized by magnetic stirring for 10 min to ensure uniform dispersion. No post-addition heat treatment was applied in order to preserve the native physicochemical and sensory characteristics of the juice.

### 2.5. Characterization of Nanoparticles

The physicochemical properties of the developed protein–β-glucan nanoparticles were evaluated in terms of hydrodynamic diameter, polydispersity index (PDI), and zeta potential using dynamic light scattering (DLS) (modified methods based on Coccia and Falcone [22] and Ahranjani and Ferrentino [23]).

Measurements were performed using a DLS instrument (Zetasizer Nano ZS, Malvern Panalytical, UK) at 25 °C. Prior to DLS analysis, the nanoparticle suspensions were diluted 50-fold (1:50) with deionized water to minimize multiple scattering effects and ensure accurate particle size and zeta potential measurements. Each sample was analyzed in triplicate, and results are reported as mean ± standard deviation.

Zeta potential was determined to assess surface charge and colloidal stability of the nanoparticles. Electrophoretic mobility was measured under an applied electric field and converted to zeta potential values using the Smoluchowski model.

The polydispersity index (PDI) was used as an indicator of particle size distribution width, where values below 0.3 were considered indicative of relatively homogeneous nanoparticle populations.

For stability assessment, nanoparticle-containing juice formulations (V3: 0.1% and V4: 0.3% *w*/*v*) were stored at 4 °C for 28 days under refrigerated conditions. The 28-day storage period was selected as a short-term refrigerated stability assessment based on the expected storage duration of minimally processed fresh juice products and to evaluate potential physicochemical changes in the nanoparticle system during cold storage. This period was not intended to define a commercial shelf life, but rather to assess the stability of nanoparticle dispersion under refrigerated conditions. Samples were maintained under controlled storage conditions, and no additional processing or agitation was applied during storage. Particle size, PDI, and zeta potential were measured at predetermined time points (0, 7, 14, and 28 days) to evaluate physicochemical stability and potential aggregation behavior over time.

### 2.6. Transmission Electron Microscopy (TEM)

The morphology and structural characteristics of the protein–β-glucan nanoparticles were examined using transmission electron microscopy (TEM). Nanoparticle suspensions obtained from formulations V3 (0.1%) and V4 (0.3%) were diluted with ultrapure water and deposited onto carbon-coated copper grids. Excess liquid was carefully removed using filter paper, and the samples were air-dried at room temperature under ambient conditions prior to analysis. TEM samples were prepared without additional negative staining to minimize potential artifacts associated with staining reagents and to preserve the native morphology of the freeze-dried nanoparticle structures. However, the absence of negative staining may reduce contrast and can influence the apparent particle boundaries; therefore, TEM images were interpreted together with DLS measurements to provide a more reliable assessment of nanoparticle size and morphology.

TEM imaging was performed at an accelerating voltage of 80–120 kV using a JEOL JEM-2100 transmission electron microscope (JEOL Ltd., Tokyo, Japan). Representative micrographs were collected at different magnifications to evaluate particle morphology, aggregation behavior, and structural uniformity [24]. The observed TEM morphology was used to qualitatively complement particle size distribution and colloidal stability data obtained by dynamic light scattering (DLS), enabling comparison between dry-state and hydrodynamic particle dimensions.

### 2.7. Fourier Transform Infrared Spectroscopy (FTIR)

Fourier Transform Infrared Spectroscopy (FTIR) was used to characterize purified β-glucan, *Fusarium venenatum* protein isolate, and freeze-dried protein–β-glucan nanoparticles obtained from formulations V3 (0.1% nanoparticles) and V4 (0.3% nanoparticles).

FTIR measurements were conducted using a PerkinElmer System 2000 FTIR spectrometer (PerkinElmer, Waltham, MA, USA) equipped with a Specac Golden Gate attenuated total reflectance (ATR) accessory. The ATR configuration was selected because it enables direct analysis of freeze-dried protein–β-glucan nanoparticle powders without additional sample preparation, such as pellet formation with potassium bromide (KBr), which may alter the native interactions between protein and polysaccharide components. ATR-FTIR is particularly suitable for complex food biopolymer systems due to its rapid measurement procedure, minimal sample manipulation, and ability to characterize functional group interactions in heterogeneous matrices. Spectra were recorded over the range of 4000–500 cm^−1^ at a resolution of 4 cm^−1^. For each sample, 32 scans were collected and averaged to improve the signal-to-noise ratio. Background spectra were recorded prior to each measurement and automatically subtracted from sample spectra.

Characteristic spectral regions associated with proteins and polysaccharides were considered during spectral analysis, including the hydroxyl stretching region (3600–3000 cm^−1^), amide I (1700–1600 cm^−1^), amide II (1580–1500 cm^−1^), and the carbohydrate fingerprint region (1200–900 cm^−1^).

### 2.8. In Vitro Gastrointestinal Digestion and Bioaccessibility of Functional Components

#### 2.8.1. Simulated Gastrointestinal Digestion Protocol

In vitro digestion of peach juice was performed according to a modified INFOGEST static digestion protocol for liquid matrices described by Brodkorb et al. [25], comprising sequential oral, gastric, and intestinal phases. The modifications primarily consisted of periodic sampling during the gastric and intestinal phases (0, 30, 60, and 120 min) to evaluate β-glucan release kinetics, bioaccessibility, protein digestibility, and antioxidant retention. In addition, enzymatic activity was terminated by heating (95 °C, 5 min) after completion of the intestinal phase to preserve the composition of the final digesta for subsequent analyses. These modifications were introduced to enable time-dependent evaluation of the functional properties of the nanoparticle-enriched peach juice and did not alter the overall sequence or physiological conditions of the INFOGEST digestion protocol.

For the oral phase, 10 mL of peach juice was mixed 1:1 (*v*/*v*) with simulated salivary fluid containing α-amylase (75 U/mL) and CaCl_2_ (1.5 mM), adjusted to pH 7.0, and incubated at 37 °C for 2 min under gentle agitation (100 rpm).

The gastric phase was initiated by adding simulated gastric fluid (1:1, *v*/*v*) containing porcine pepsin (2000 U/mL) and CaCl_2_ (0.15 mM), adjusted to pH 3.0. Samples were incubated at 37 °C for 120 min under continuous agitation. Aliquots (1 mL) were collected at 0, 30, 60, and 120 min, immediately cooled on ice, and stored at −20 °C until analysis. Enzymatic activity was maintained during sampling without intermediate inactivation.

For the intestinal phase, simulated intestinal fluid (1:1, *v*/*v*) containing pancreatin (trypsin activity 100 U/mL) and bile salts (10 mM) was added. CaCl_2_ concentration was adjusted to 0.6 mM, and pH was set to 7.0. Samples were incubated at 37 °C for 120 min with sampling at 0, 30, 60, and 120 min. Enzymatic reactions were terminated by heating at 95 °C for 5 min, followed by rapid cooling on ice.

#### 2.8.2. Determination of β-Glucan Bioaccessibility

β-Glucan content was determined using a commercial enzymatic assay kit specific for mixed-linkage β-glucans (Megazyme, β-Glucan Assay Kit (Mixed Linkage), K-BGLU; SKU 700004269, Megazyme, Bray, Ireland), according to the manufacturer’s instructions [26]. Prior to analysis, samples were centrifuged at 10,000× *g* for 10 min to remove particulate matter.

Bioaccessibility was calculated as:

β-Glucan bioaccessibility (%) = (β-glucan content after digestion/initial β-glucan content) × 100 (1)

Results were expressed as mg β-glucan per 100 mL of juice and as percentage bioaccessibility.

#### 2.8.3. Protein Digestibility

Protein digestibility was determined using the o-phthaldialdehyde (OPA) assay, which quantifies free amino groups released during digestion [27]. Aliquots from gastric and intestinal phases were centrifuged at 10,000× *g* for 10 min prior to analysis, and supernatants were reacted with OPA reagent. Absorbance was measured at 340 nm.

Protein digestibility was calculated as follows:

Protein digestibility (%) = (free amino groups after digestion/total protein content) × 100 (2)

Results were expressed as percentage digestibility relative to initial protein concentration.

#### 2.8.4. Antioxidant Retention During Digestion

Antioxidant capacity was evaluated before digestion and after the intestinal phase using DPPH and FRAP assays, as previously described [20,28]. Antioxidant retention was calculated as follows:

Antioxidant retention (%) = (antioxidant activity after digestion/initial antioxidant activity) × 100 (3)

Results were expressed as μmol Trolox equivalents (TE) per 100 mL and as retention percentage.

#### 2.8.5. Release Kinetics During Digestion

The release kinetics of β-glucans and antioxidant compounds during gastric and intestinal digestion were modeled using concentration data collected at 0, 30, 60, and 120 min. A first-order kinetic model was applied:

C_t_ = C_0_ (1 − e^−kt^) (4)

where C_t_ is the concentration at time t; C_0_ is the maximum releasable concentration; k is the release rate constant (min^−1^).

Nonlinear regression analysis was performed using SPSS 30 (IBM Corp., Armonk, NY, USA), and goodness-of-fit was evaluated using the coefficient of determination (R^2^). The area under the curve (AUC) was calculated using the trapezoidal rule to describe the overall release profile during digestion.

### 2.9. Metabolic Health Proxies

#### 2.9.1. α-Amylase Inhibition Assay

The inhibitory effect of peach juice formulations (V1–V4) on α-amylase activity was evaluated using a colorimetric assay based on porcine pancreatic α-amylase-mediated hydrolysis of soluble starch and subsequent quantification of reducing sugars. Porcine pancreatic α-amylase (≥10 U/mg) was prepared in 20 mM phosphate buffer (pH 6.9) containing 6 mM NaCl to a final activity of 1 U/mL, while 1% (*w*/*v*) soluble starch in the same buffer served as substrate.

Juice samples were centrifuged at 10,000× *g* for 10 min and used directly in the assay. Serial dilutions (20–100%) were prepared for IC_50_ determination. For the assay, 250 μL of enzyme solution was pre-incubated with 250 μL of sample at 37 °C for 10 min, followed by addition of 250 μL starch solution. After incubation for 15 min at 37 °C, the reaction was terminated by adding 500 μL of freshly prepared DNS reagent containing 1% (*w*/*v*) 3,5-dinitrosalicylic acid, 1% (*w*/*v*) NaOH, and 30% (*w*/*v*) potassium sodium tartrate, followed by heating at 95 °C for 5 min, cooling, dilution with 5 mL distilled water, and absorbance measurement at 540 nm [29,30].

α-Amylase inhibition (%) was calculated as follows:

α-Amylase inhibition (%) = (1 − A_sample_/A_control_) × 100 (5)

where A_control_ is the absorbance of the control reaction without sample, and A_sample_ is the absorbance in the presence of the juice sample.

IC_50_ values were determined by plotting inhibition percentage against sample concentration and fitting the data to a nonlinear regression model.

#### 2.9.2. α-Glucosidase Inhibition Assay

α-Glucosidase inhibitory activity was determined using a p-nitrophenyl-α-D-glucopyranoside (pNPG) assay, which quantifies the release of p-nitrophenol. α-Glucosidase from *Saccharomyces cerevisiae* (≥10 U/mg) was prepared in 100 mM phosphate buffer (pH 6.8) to a final activity of 1 U/mL, and pNPG substrate was prepared at 5 mM in the same buffer.

In a 96-well microplate, 50 μL of sample was mixed with 50 μL enzyme solution and pre-incubated at 37 °C for 10 min. Subsequently, 50 μL substrate solution was added, and the mixture was incubated for 20 min at 37 °C. The reaction was stopped by adding 100 μL of 0.1 M Na_2_CO_3_, and absorbance was measured at 405 nm [29,30].

α-Glucosidase inhibition (%) was calculated as follows:

α-Glucosidase inhibition (%) = (1 − A_sample_/A_control_) × 100 (6)

IC_50_ values were determined using the same nonlinear regression approach described for α-amylase inhibition.

#### 2.9.3. Evaluation of Synergistic Inhibition Effects

To evaluate potential interaction effects between protein and β-glucan components in the juice formulations, a theoretical additive model was applied [31]. The expected inhibitory effect was calculated assuming independent additive contributions of the individual components:

E_expected_ = E_protein_ + E_β-glucan_ (7)

where E_protein_ and E_β-glucan_ represent the individual inhibitory effects (%) determined under identical assay conditions using the corresponding isolated or control systems.

This model assumes no interaction between components and is widely used as a first-order approximation for synergy assessment in food systems.

To account for potential overlap between mechanisms of action, the experimentally observed inhibition of the whole nanoparticle-enriched juice formulations (V3 and V4) was compared with the theoretical additive response using a synergy index (SI):

SI = E_expected_/E_observed_ (8)

where E_observed_ represents the experimentally measured inhibition of the complete formulation.

SI values > 1 indicate potential synergistic interactions, SI = 1 indicates additive effects, and SI < 1 suggests antagonistic or non-additive interactions.

### 2.10. Sensory Analysis

A total of 60 untrained participants (40 women and 20 men, aged 25–60 years) evaluated four peach juice variants (≈30 mL, 10 ± 2 °C) under controlled laboratory conditions. Participants were recruited as regular consumers and provided informed consent prior to participation. Samples were presented in transparent cups following a standardized randomized tasting protocol for liquid products to minimize order effects. Facial expressions during tasting were recorded at 1080 × 720 pixel resolution and 30 frames per second under controlled overhead lighting conditions. A fixed camera position and adjustable head-support system were used to ensure consistent face positioning and recording quality.

Video recordings were analyzed using iMotions 6.2 software [32] with the Affectiva engine, extracting emotional expression parameters. In parallel, hedonic acceptability was assessed using a nine-point hedonic scale (1 = “dislike extremely,” 5 = neutral, 9 = “like extremely”).

The analysis was designed to evaluate overall consumer acceptability, perceived sensory quality, and the relationship between conventional hedonic scoring and digitally extracted facial emotional responses [28].

### 2.11. Multivariate Analysis of Formulation-Performance Relationships

Experimental data including β-glucan bioaccessibility, protein digestibility, antioxidant retention, α-amylase and α-glucosidase inhibition, and sensory scores were used to evaluate relationships between formulation characteristics and functional performance. Nanoparticle concentration, β-glucan content, protein content, release rate constant (k), and storage time were used as predictor variables.

Regression-based modeling was performed using SPSS 30 (IBM Corp., Armonk, NY, USA) to estimate the effects of formulation variables on α-amylase and α-glucosidase inhibitory activity. Predicted IC50 values were generated for each formulation and storage condition using the selected predictor variables. Model performance was evaluated using the coefficient of determination (R^2^) to assess the agreement between predicted and observed responses.

### 2.12. Statistical Analysis

Differences among the four juice formulations (V1–V4) were analyzed using one-way analysis of variance (ANOVA), followed by Tukey’s HSD post hoc test for multiple pairwise comparisons. Independent-samples *t*-tests were used for comparisons between two groups. Statistical significance was set at *p* < 0.05. The assumptions of normality and homogeneity of variance were verified prior to ANOVA.

Principal component analysis (PCA) was performed to visualize patterns and clustering among samples based on functional and sensory parameters. Prior to PCA, variables were standardized using z-score normalization. All statistical analyses were conducted using SPSS version 30 (IBM Corp., Armonk, NY, USA).

## 3. Results

### 3.1. Physicochemical Characterization of Nanoparticles

The physicochemical properties of the protein–β-glucan nanoparticles incorporated into peach juice formulations (V3 and V4) are summarized in Table 1. Both formulations exhibited particle sizes within the nanoscale range, with mean diameters of 176.25 ± 11.52 nm for V3 and 228.74 ± 14.91 nm for V4. A significantly higher particle size (*p* < 0.05) was observed in V4 compared with V3.

All formulations showed polydispersity index (PDI) values below 0.30, indicating a relatively narrow size distribution and acceptable homogeneity of the colloidal systems. The zeta potential values were negative for both formulations, measuring −19.63 mV (V3) and −23.84 mV (V4), indicating the presence of negatively charged surface functional groups.

#### 3.1.1. Stability During Storage

The stability of the protein–β-glucan nanoparticle systems during refrigerated storage (4 °C for 28 days) is presented in Table 2. Both formulations exhibited a time-dependent increase in particle size and polydispersity index (PDI) throughout the storage period.

After 28 days, particle size increased significantly (*p* < 0.05) to 191.36 nm for V3 and 248.93 nm for V4. A gradual increase in PDI was also observed in both formulations over time. Zeta potential values showed a progressive decrease in magnitude during storage, reaching −18.04 mV (V3) and −21.63 mV (V4) at day 28.

All measured parameters changed significantly over the storage period (*p* < 0.05), as indicated in Table 2.

#### 3.1.2. Morphological Characterization of Protein–β-Glucan Nanoparticles by TEM

**Figure 1:**
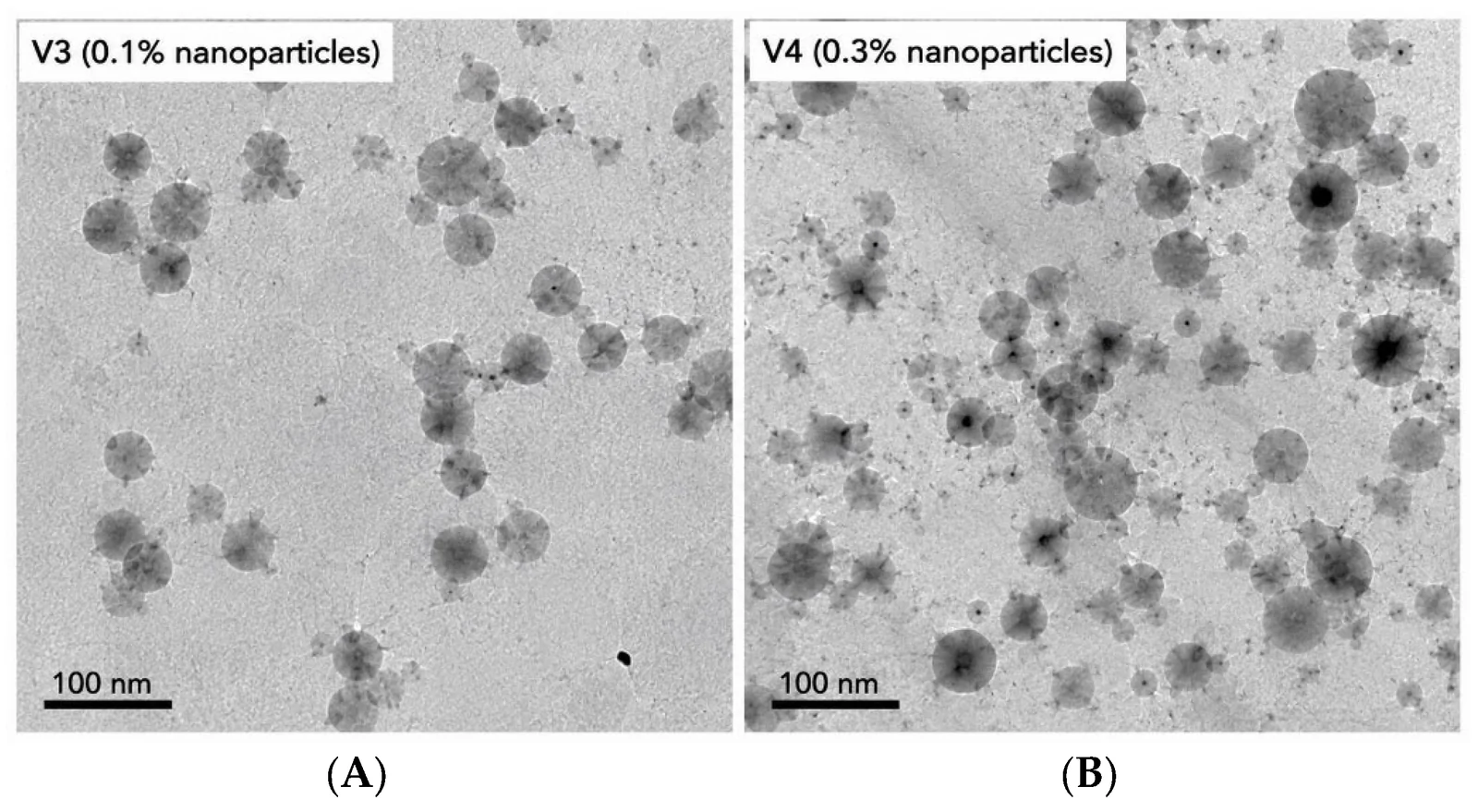
Transmission electron microscopy (TEM) micrographs of protein–β-glucan nanoparticles incorporated into peach juice formulations: (**A**) V3 containing 0.1% nanoparticles and (**B**) V4 containing 0.3% nanoparticles. Predominantly spherical to near-spherical nanoparticles with moderate size variability were observed. The higher particle density observed in V4 reflects the increased nanoparticle concentration, while differences in apparent particle dimensions may be influenced by TEM sample preparation and drying effects. Scale bar = 100 nm.

Representative TEM micrographs of protein–β-glucan nanoparticles are shown in Figure 1. Both formulations exhibited predominantly spherical to near-spherical morphology with smooth contours, confirming successful formation of nanoscale structures via protein–polysaccharide self-assembly.

V3 (0.1%) showed a more dispersed distribution with clearly distinguishable individual nanoparticles, whereas V4 (0.3%) exhibited higher particle density and localized clustering, consistent with increased nanoparticle concentration. Although some individual particles in the V4 micrographs appeared smaller than those observed in V3, this difference should be interpreted cautiously because TEM provides measurements of dehydrated particles after drying on the grid, whereas DLS determines the hydrodynamic diameter of hydrated particles dispersed in the juice matrix. The apparent variation in particle dimensions in TEM images may therefore reflect differences in drying behavior, particle orientation, and local distribution rather than a true reduction in nanoparticle size. No extensive aggregation was observed, indicating maintained colloidal stability.

Particle dimensions observed by TEM were generally lower than those obtained by DLS, which is expected due to analysis of dehydrated particles in TEM versus hydrodynamic diameters in DLS measurements. Moderate polydispersity observed in TEM was consistent with PDI values, confirming controlled size distribution.

Overall, TEM analysis confirmed the successful formation of stable protein–β-glucan nanoparticles suitable for incorporation into functional peach juice systems.

### 3.2. FTIR Characterization of Protein–β-Glucan Nanoparticles

**Figure 2:**
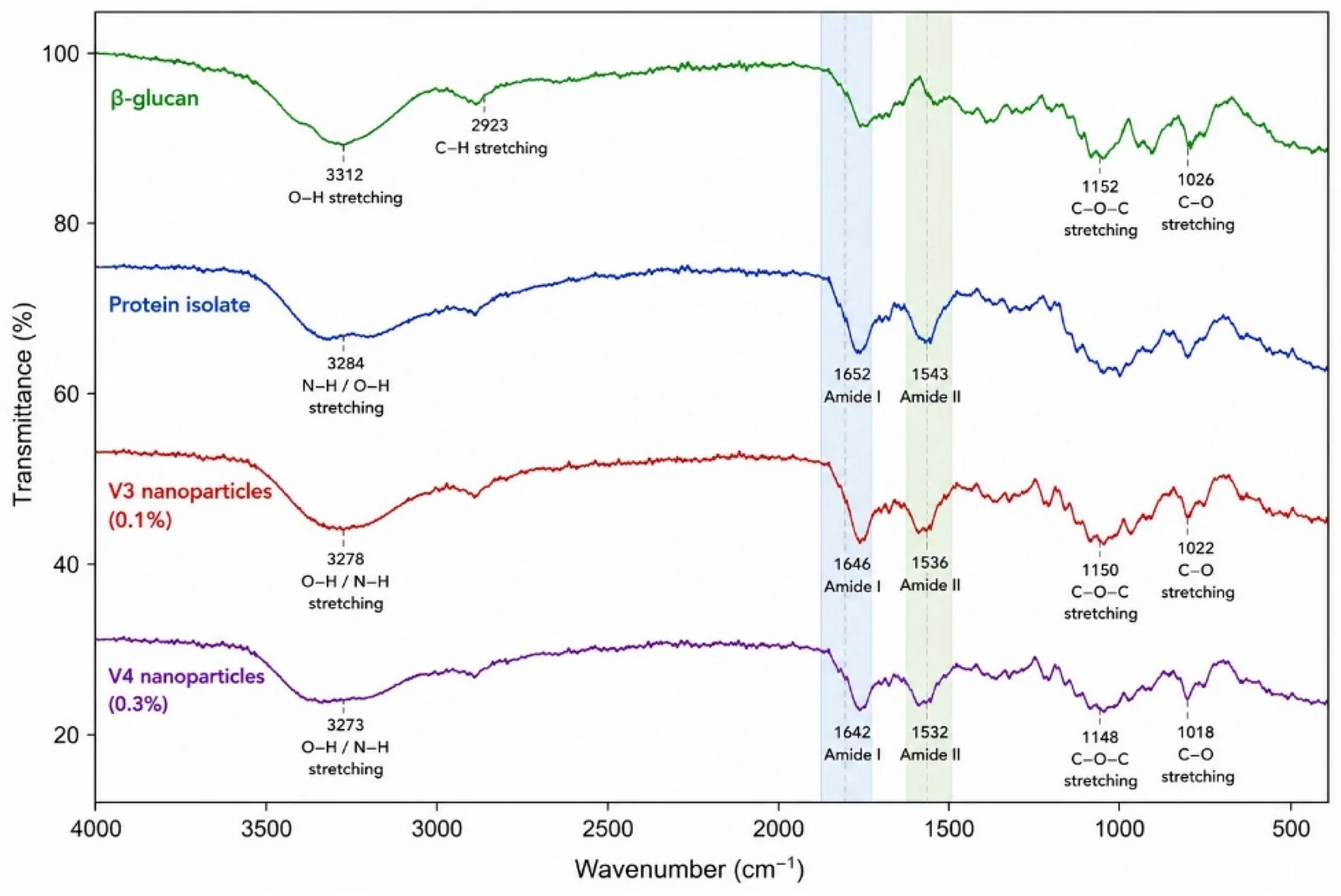
FTIR spectra of β-glucan, protein isolate, and protein–β-glucan nanoparticles (V3 and V4). The observed shifts in amide I and amide II bands together with broadening of the hydroxyl stretching region indicate intermolecular interactions between protein and β-glucan components during nanoparticle formation.

The FTIR spectra of β-glucan, protein isolate, and protein–β-glucan nanoparticles are presented in Figure 2. The β-glucan spectrum exhibited a broad absorption band at ~3312 cm^−1^ corresponding to O–H stretching vibrations, along with characteristic polysaccharide bands in the 1152–1026 cm^−1^ region attributed to C–O–C and C–O stretching vibrations.

The protein isolate showed characteristic amide I and amide II bands at ~1652 and 1543 cm^−1^, respectively, associated with protein secondary structure. A broad band at ~3284 cm^−1^ was also observed, assigned to overlapping N–H and O–H stretching vibrations.

After nanoparticle formation, both V3 and V4 retained the characteristic spectral features of protein and β-glucan. However, shifts in the amide I band from 1652 cm^−1^ to 1646 cm^−1^ (V3) and 1642 cm^−1^ (V4), as well as in the amide II band from 1543 cm^−1^ to 1536 and 1532 cm^−1^, respectively, were observed. In addition, broadening of the O–H stretching region was detected in both nanoparticle formulations.

These spectral changes are consistent with the occurrence of intermolecular interactions between protein and β-glucan components during nanoparticle formation. However, FTIR analysis alone does not allow direct identification of specific interaction types or complete elucidation of molecular assembly mechanisms. The polysaccharide fingerprint region remained unchanged, confirming preservation of β-glucan structural integrity. No new absorption bands associated with covalent bond formation were detected, suggesting that the observed structural organization is consistent with non-covalent interactions commonly reported for protein–polysaccharide systems.

Overall, FTIR analysis indicates interactions between protein and β-glucan components while maintaining the structural characteristics of both biopolymer components.

### 3.3. Microstructure and Physical Stability of Juice Matrix

The viscosity and turbidity of peach juice formulations are presented in Table 3. Viscosity values ranged from 2.18 to 3.12 mPa·s across the formulations, with the lowest value observed in V2 and the highest in V4. Turbidity showed a similar trend, increasing from 28.74 NTU (V2) to 78.92 NTU (V4).

Commercial juice (V1) exhibited intermediate values for both parameters, while nanoparticle-enriched formulations (V3 and V4) showed higher viscosity and turbidity compared to V1 and V2. All differences among formulations were statistically significant (*p* < 0.05).

### 3.4. In Vitro Gastrointestinal Digestion and Bioaccessibility of Functional Components

The β-glucan content and bioaccessibility during in vitro gastrointestinal digestion are presented in Table 4. Initial β-glucan concentrations ranged from 5.35 to 40.27 mg/100 mL across formulations, with significantly higher values (*p* < 0.05) observed in nanoparticle-enriched samples (V3 and V4) compared to control variants (V1 and V2).

During the gastric phase, β-glucan levels ranged from 3.41 to 29.95 mg/100 mL, while gastric bioaccessibility varied between 63.72% and 74.45%. The highest gastric bioaccessibility was recorded in V4. In the intestinal phase, β-glucan concentrations increased across all formulations, with values between 4.59 and 35.02 mg/100 mL, corresponding to intestinal bioaccessibility of 83.67% to 87.09%.

Protein digestibility values are presented in Table 5. In the gastric phase, digestibility ranged from 30.88% (V3) to 36.24% (V4), while in the intestinal phase values ranged from 69.74% to 75.46% across formulations. Significant differences (*p* < 0.05) were observed among all variants in both digestion phases.

Antioxidant activity and retention during in vitro gastrointestinal digestion are presented in Table 6. DPPH values ranged from 14.78 to 25.78 μmol TE/100 mL across formulations, with a progressive increase observed from V1 to V4. All variants showed statistically significant differences (*p* < 0.05).

FRAP values ranged from 12.23 to 23.50 μmol TE/100 mL. No significant differences were observed for FRAP absolute values between all formulations. FRAP retention values ranged from 79.01% to 83.96%, with significantly higher values recorded in nanoparticle-enriched formulations (V3 and V4) compared to control samples (V1 and V2).

β-Glucan bioaccessibility and release kinetics during in vitro gastrointestinal digestion are presented in Table 7. Initial β-glucan concentrations ranged from 5.35 to 40.27 mg/100 mL across formulations, with significantly higher values observed in nanoparticle-enriched samples (V3 and V4) compared to control variants (V1 and V2) (*p* < 0.05).

β-Glucan bioaccessibility values ranged from 83.66% to 86.97% across all formulations. Release rate constants (k) varied between 0.021 and 0.027 min^−1^. The highest k value was observed in V4, while the lowest values were recorded in V1 and V3. AUC values increased progressively from 43.97 to 349.87 mg·min/100 mL across the formulations.

The first-order kinetic model showed R^2^ values ranging from 0.942 to 0.971 across all formulations.

### 3.5. Metabolic Health Proxies

α-Amylase inhibitory activity and IC_50_ values of the peach juice formulations are presented in Table 8. Inhibition ranged from 32.47% to 45.36% across the formulations, with the lowest value observed in V1 and the highest in V4. Significant differences (*p* < 0.05) were observed among all variants.

IC_50_ values ranged from 72.34 mg/mL (V1) to 53.87 mg/mL (V4), with intermediate values observed for V2 and V3.

α-Glucosidase inhibitory activity and IC_50_ values of the peach juice formulations are presented in Table 9. Inhibition ranged from 28.63% to 43.48% across the formulations, with the lowest value observed in V1 and the highest in V4. Significant differences (*p* < 0.05) were observed among all variants.

Synergy analysis based on the Bliss independence model demonstrated a progressive increase in synergy index (SI) values with increasing nanoparticle concentration, indicating enhanced cooperative interactions between protein and β-glucan fractions.

IC_50_ values ranged from 79.25 mg/mL (V1) to 56.39 mg/mL (V4), with intermediate values observed for V2 and V3.

Synergy Index (SI) values ranged from 0.98 to 1.15 across formulations, with the lowest value in V1 and the highest in V4.

### 3.6. Sensory Evaluation and Consumer Preference

The sensory evaluation results (Table 10) revealed significant differences among the peach juice variants (*p* < 0.05) across all assessed attributes. Appearance liking increased progressively from V1 (6.12) to V4 (7.82), with V3 and V4 showing higher scores compared to the control formulations. Taste liking ranged from 5.98 (V1) to 7.52 (V3), while V4 showed a slightly lower value (6.74). Mouthfeel liking followed an increasing trend from V1 (5.87) to V4 (7.69). Overall liking was highest for V4 (8.77) and V3 (8.53), with no significant difference between these two variants (*p* > 0.05). Purchase intention increased from 62.32% (V1) to 78.59% (V4), with all variants differing significantly (*p* < 0.05).

Digital sensory analysis (Table 11) showed consistent trends with conventional sensory evaluation. Lip press values decreased from V1 (0.42) to V4 (0.34), while lip suck decreased from 0.31 to 0.26. Mouth open increased across formulations, ranging from 0.47 (V1) to 0.54 (V4). Head orientation parameters (yaw and roll) showed a gradual decrease across variants, with the lowest values observed in V4.

In terms of emotional response, joy increased from 0.62 (V1) to 0.70 (V4), while disgust decreased from 0.12 to 0.09. Relaxed expression increased progressively across formulations. Emoji-based responses showed an increase in “smiley” expression from 0.58 (V1) to 0.67 (V4), while “smirk” decreased from 0.32 to 0.27. Other emoji parameters showed minor or non-significant variations.

### 3.7. Multivariate Analysis of Formulation-Performance Relationships

The predictive model for functional properties (Table 12) indicates that β-glucan concentration, protein content, release rate constant (k), and storage time collectively influence the predicted inhibitory activity against α-amylase and α-glucosidase. A consistent decrease in predicted IC_50_ values is observed with increasing β-glucan content across all formulations. In this context, V4 (≈40 mg/100 mL β-glucan) shows the lowest predicted IC_50_ values for both enzymes, whereas V1 exhibits the highest values.

Storage time (0 vs. 28 days) results in a slight reduction in predicted IC_50_ values across all variants, indicating minor temporal variation in model outputs. The effect of protein content is more pronounced in formulations with higher β-glucan levels (V3 and V4), where lower predicted IC_50_ values are consistently observed compared to low-β-glucan variants.

The regression-based predictive models demonstrated good agreement between variables, with coefficients of determination of R^2^ = 0.912 for α-amylase and R^2^ = 0.894 for α-glucosidase inhibition models, respectively.

**Figure 3:**
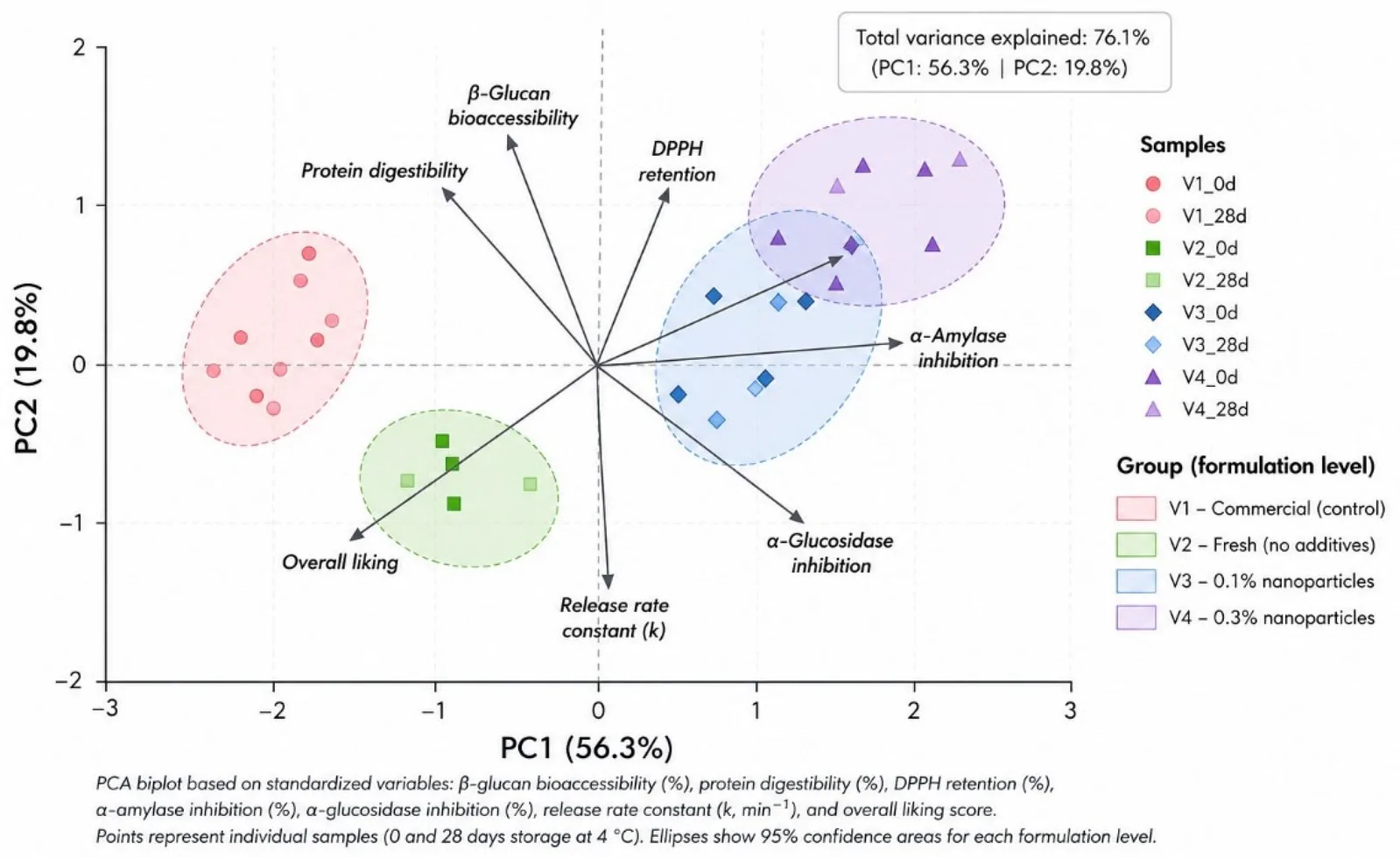
PCA of functional, metabolic and sensory attributes of peach juice formulations.

Principal component analysis (PCA) was performed to evaluate relationships among functional, metabolic, compositional, and sensory parameters of the peach juice formulations (Figure 3). The analysis included standardized variables describing β-glucan bioaccessibility, protein digestibility, antioxidant retention, enzyme inhibitory activity, release kinetics, and sensory acceptability.

The first two principal components (PC1 and PC2) explained 76.1% of the total variance, with PC1 accounting for 56.3% and PC2 for 19.8%. This indicates that the selected variables sufficiently captured the major variability among formulations.

PC1 was primarily associated with functional and bioactive properties, with strong positive loadings of α-amylase inhibition, α-glucosidase inhibition, DPPH retention, and β-glucan bioaccessibility. Accordingly, nanoparticle-enriched formulations (V3 and V4) were positioned on the positive side of PC1, while control formulations (V1 and V2) clustered on the negative side, reflecting lower functional performance.

PC2 described secondary variation related to compositional and sensory-related responses. Positive loadings included protein digestibility and β-glucan bioaccessibility, whereas release rate constant (k) and overall liking showed opposite loading patterns. No clear separation of samples according to storage time was observed, indicating that refrigerated storage for up to 28 days induced limited shifts in the multivariate profile.

Partial overlap between V3 and V4 was observed in the score plot, indicating similar multivariate behavior. However, V4 was more closely associated with enzyme inhibitory activity and antioxidant retention, while V3 showed a relatively stronger association with sensory-related variables. Overall, PCA indicated that nanoparticle incorporation was the primary factor driving differentiation among formulations, with secondary variation reflecting compositional balance between functional and sensory attributes.

## 4. Discussion

The obtained results confirm the successful formation of protein–β-glucan-based colloidal nanoparticles within the peach juice matrix, exhibiting physicochemical characteristics consistent with food-grade nanosystems. Particle size increased from approximately 170 nm in V3 to 250 nm in V4, suggesting that increasing nanoparticle concentration enhanced protein–β-glucan intermolecular interactions and promoted the formation of larger, more compact colloidal assemblies [33,34]. Similar concentration-dependent behavior has been reported by Yang and Cheung [35] and Kaur et al. [36], who likewise attributed particle growth to intensified biopolymer interactions.

To the best of our knowledge, limited information is available regarding the application of thermally induced protein–β-glucan nanoparticles based on *Fusarium venenatum* protein isolate and *Fuscoporia torulosa* β-glucans in fruit beverage systems. The present study extends previous research on protein–polysaccharide delivery systems by evaluating their combined effects on physicochemical stability, gastrointestinal bioaccessibility, functional properties, and sensory performance within a peach juice matrix.

PDI values below 0.30 indicate a relatively narrow particle-size distribution and satisfactory colloidal homogeneity, which is considered acceptable for complex food dispersions where complete monodispersity is rarely achieved. Both formulations exhibited zeta potential values below −30 mV, with V4 showing a more negative surface charge, indicating enhanced electrostatic stabilization at higher nanoparticle concentrations. Increased electrostatic repulsion between protein–β-glucan complexes has previously been associated with reduced particle aggregation and improved colloidal stability [37,38]. However, the measured zeta potential values should not be interpreted as evidence of complete colloidal stability, as electrostatic repulsion represents only one of the factors governing particle stability in complex food matrices. The observed behavior may also be influenced by particle concentration, biopolymer interactions, and the presence of other components in the dispersion, which can affect particle–particle interactions and promote aggregation despite differences in surface charge. The greater negative surface charge observed for V4 may be attributed to increased exposure of anionic functional groups resulting from structural rearrangement at higher biopolymer concentrations.

Storage stability analysis showed that both formulations remained relatively homogeneous over 28 days at 4 °C. Although gradual increases in particle size and PDI, together with slight changes in zeta potential, suggested limited structural rearrangement during storage, these changes may be associated with the slow hydration of β-glucan chains and progressive protein–polysaccharide reorganization typically observed in acidic fruit-based systems rather than irreversible aggregation [39]. The persistence of relatively low PDI values and negative zeta potential throughout storage suggests that particle aggregation was not extensive; however, these parameters alone do not provide definitive evidence of colloidal stability in the complex juice matrix. The observed changes may reflect the combined effects of electrostatic and non-electrostatic interactions within the protein–β-glucan system during refrigerated storage [40,41].

TEM analysis corroborated the DLS and zeta potential results by providing direct visual evidence of protein–β-glucan nanoparticle formation. Both formulations exhibited predominantly spherical to near-spherical nanoparticles with smooth contours, confirming the successful self-assembly of the protein–β-glucan complexes. Similar morphologies have been widely reported for β-glucan-based particulate systems, supporting the reproducibility of this self-assembly behavior across different biopolymer formulations [42,43,44,45]. The higher particle density and occasional clustering observed in V4 are consistent with its higher nanoparticle concentration and may explain the larger hydrodynamic diameters measured by DLS. However, the smaller particle dimensions observed by TEM compared with DLS are expected because TEM measures dehydrated particles, whereas DLS determines the hydrodynamic diameter of hydrated particles in suspension; therefore, direct size comparisons should be interpreted with caution. The absence of extensive aggregation is consistent with the highly negative zeta potential values and low PDI, suggesting that electrostatic repulsion may have contributed to limiting particle aggregation. However, zeta potential and PDI values alone do not provide definitive evidence of colloidal stability, particularly in a complex food-based system where other interactions may also influence particle association. Because negative staining was not applied, particle boundaries may not have been fully resolved, and the TEM observations should therefore be regarded as complementary to DLS rather than as quantitative particle-size measurements [46].

The increase in viscosity and turbidity observed after nanoparticle incorporation reflects the formation of a structured colloidal network within the peach juice matrix. The higher viscosity values in V3 and V4 suggest enhanced interactions between protein–β-glucan nanoparticles and native juice constituents, resulting in increased resistance to flow and weak structuring effects characteristic of protein–polysaccharide dispersions. Similar viscosity-enhancing behavior has been reported for β-glucan–protein systems, where intermolecular associations contribute to network formation and improved dispersion stability [37,38,47]. The increased turbidity is attributed to enhanced light scattering by dispersed colloidal particles, which is consistent with the nanoscale structures observed by DLS and TEM. Despite these changes, the formulations remained within the viscosity range expected for beverage applications, indicating that nanoparticle incorporation improved structural properties without compromising technological feasibility. From a clean-label perspective, the use of protein–β-glucan nanoparticles represents a potential strategy for improving beverage stability without synthetic stabilizers; however, further studies involving extended shelf-life evaluation, microbial stability, and oxidative stability are required before their application as complete replacements for conventional additives.

FTIR analysis further supported the formation of protein–β-glucan nanoparticles and complemented the structural observations obtained from TEM, DLS, and zeta potential measurements. ATR-FTIR analysis of freeze-dried nanoparticles allowed characterization of molecular interactions while minimizing extensive sample preparation that could potentially alter protein–polysaccharide associations. The retention of characteristic absorption bands from both biopolymers indicates that nanoparticle formation occurred without major disruption of their chemical structures [48]. The observed shifts in the amide I and amide II regions, together with broadening of the O–H stretching band, suggest alterations in the local environment of protein and polysaccharide functional groups, which are compatible with intermolecular interactions such as hydrogen bonding and electrostatic associations. However, FTIR provides indirect structural information and cannot independently define the exact molecular arrangement or interaction energies within the nanoparticle system. Similar spectral behavior has been reported for β-glucan-based nanoparticle systems, where changes in hydroxyl and glycosidic regions were associated with altered hydrogen-bonding interactions and exposure of functional groups following nanoscale assembly [49,50,51].

The more pronounced spectral changes observed in V4 suggest stronger intermolecular associations at higher nanoparticle concentrations. The absence of additional absorption bands indicates that nanoparticle formation was primarily governed by non-covalent interactions rather than covalent modification, which is consistent with the behavior of food-grade protein–polysaccharide systems. Preservation of the characteristic β-glucan fingerprint region (1150–1020 cm^−1^) confirms the maintenance of the polysaccharide backbone, supporting the retention of β-glucan-related functional properties. Overall, the FTIR results indicate successful incorporation of β-glucan within the protein matrix while maintaining the chemical identity of both biopolymers, supporting their role as natural structuring components in functional beverage formulations.

From a gastrointestinal digestion perspective, nanoparticle incorporation enhanced the apparent β-glucan bioaccessibility and release behavior of the formulations. The detectable β-glucan levels observed in the control samples (V1 and V2) may be associated with background signals originating from the complex fruit matrix or non-specific interactions during enzymatic analysis; therefore, these values should be interpreted as apparent assay-derived β-glucan content rather than evidence of naturally occurring β-glucan in peach juice. Further structural analyses would be required to distinguish specific β-glucan signals from potential matrix-related responses.

High intestinal β-glucan bioaccessibility (>83%) was observed in all nanoparticle-enriched formulations, with V4 showing the highest release performance. This behavior suggests that increasing nanoparticle concentration enhanced the availability of β-glucan during simulated digestion, possibly through improved protection of the polysaccharide within the protein matrix. Protein–polysaccharide nanoparticles may limit premature degradation during gastric digestion while facilitating progressive release under intestinal conditions, thereby increasing the soluble fraction available for subsequent interactions. Similar controlled-release mechanisms have been described for protein-based food delivery systems.

The slightly lower intestinal protein digestibility observed for V3 compared with V1 and V2 may reflect the formation of a more compact protein–β-glucan network that temporarily reduced enzyme accessibility. In contrast, the higher nanoparticle concentration in V4 may have induced structural rearrangements that improved substrate accessibility during digestion. However, this interpretation remains speculative and requires confirmation through additional structural and enzymatic studies. The higher protein digestibility observed in V4 further suggests that protein–β-glucan interactions did not prevent enzymatic hydrolysis and may contribute to improved digestion behavior, consistent with previous findings on protein–polysaccharide systems [9,26].

The enzymatic assay provided quantitative estimation of β-glucan content; however, detailed characterization of the fungal β-glucan structure, including monosaccharide composition and linkage analysis, was not performed. Future studies should therefore include advanced structural approaches to confirm molecular composition and establish structure–function relationships. Although microbial-derived β-glucans and proteins represent promising functional ingredients, further evaluation of safety aspects, including allergenicity and regulatory suitability, remains necessary for future food applications.

Nanoparticle incorporation also improved the retention of antioxidant activity during digestion. The higher DPPH activity and FRAP retention observed in V3 and V4 indicate that the protein–β-glucan matrix provided a protective environment for bioactive compounds, potentially reducing oxidative degradation under gastrointestinal conditions. These results support the potential of protein–β-glucan nanoparticles as functional carriers capable of improving antioxidant stability and availability during digestion [52,53].

Digestive enzyme inhibition demonstrated a clear concentration-dependent effect, with nanoparticle-enriched formulations showing increased α-amylase and α-glucosidase inhibitory activity compared with control samples. This behavior may be attributed to the combined effects of β-glucan and protein components within the nanoparticle matrix. β-Glucans can increase solution viscosity and potentially reduce enzyme–substrate interactions, while protein–polysaccharide associations may influence the accessibility and interaction of bioactive components with digestive enzymes. The observed synergistic index further supports cooperative effects between the two biopolymer components, consistent with previously reported mechanisms in protein–polysaccharide systems [8,54,55]. In addition, the nanoscale organization of the particles and the release of protein-derived peptides during digestion may contribute to enhanced interactions with digestive enzymes. Although further mechanistic studies, including enzyme kinetic analysis and molecular-level characterization, are required, the present findings suggest that nanoparticle assembly can enhance the biological functionality of the individual components.

Sensory evaluation demonstrated that nanoparticle incorporation improved functional properties without compromising consumer acceptance. Formulations V3 and V4 achieved the highest overall liking scores, with V3 showing slightly higher acceptance than V4 despite its lower nanoparticle concentration. This observation suggests the existence of an optimal formulation range in which enhanced functionality is balanced with desirable sensory characteristics. Similar sensory outcomes have been reported by Aamer and El-Kholy [56] and Khoshdouni Farahani et al. [57,58], who highlighted the importance of maintaining an appropriate balance between functional ingredient incorporation and organoleptic quality. Digital sensory analysis further supported these findings by showing increased positive emotional responses, including joy, relaxation, and smile-related expressions, together with reduced negative responses such as lip pressing and disgust, indicating consistent consumer perception across complementary sensory approaches [28,59]. Importantly, nanoparticle incorporation did not negatively affect acceptance despite increases in viscosity and turbidity, suggesting that the structural modifications remained within an acceptable range for fruit beverage applications and may have contributed positively to mouthfeel characteristics [30].

PCA provided an integrated overview of the relationships among physicochemical, functional, and sensory variables and demonstrated clear differentiation between nanoparticle-enriched and control formulations. Nanoparticle incorporation represented the main factor associated with formulation separation, with V3 and V4 showing strong associations with functional parameters, including antioxidant retention, enzyme inhibition, and β-glucan bioaccessibility, whereas control formulations were positioned closer to lower bioactivity characteristics. The partial overlap between V3 and V4 indicates that increasing nanoparticle concentration did not result in strictly linear improvements across all measured responses, highlighting the importance of formulation optimization rather than simply increasing nanoparticle content.

The close association of nanoparticle-enriched formulations with multiple functional variables suggests that protein–β-glucan self-assembly simultaneously influenced several interconnected quality attributes of the beverage system. Storage-related samples remained within the same multivariate region, indicating that refrigerated storage induced only limited changes in the overall response profile. Nevertheless, the observed shifts in particle characteristics, compositional variables, and sensory parameters suggest that gradual structural rearrangements may occur during storage, requiring further evaluation through extended stability studies.

Previous studies have mainly investigated either the physicochemical characteristics of β-glucan-based nanoparticles or their individual functional properties, with limited attention given to their performance within complex beverage matrices [60]. The present study expands this knowledge by evaluating protein–β-glucan nanoparticles in a real food system, integrating colloidal characterization, gastrointestinal bioaccessibility [61], digestive enzyme inhibition [62], antioxidant retention, and digital sensory profiling [63]. This integrated approach demonstrates that these nanoparticles can simultaneously contribute to beverage stability, functional performance, and consumer acceptance, highlighting their potential as multifunctional ingredients for clean-label beverage development.

Although FTIR analysis supported the occurrence of protein–β-glucan interactions, the precise molecular mechanisms governing nanoparticle assembly and functional behavior remain to be fully elucidated. Future studies combining molecular modeling, nuclear magnetic resonance spectroscopy, and detailed enzyme kinetic analyses could provide deeper insights into the nature of protein–polysaccharide interactions, structural organization, and the mechanisms underlying the observed functional effects.

Despite these promising findings, several limitations should be considered. The biological functionality of the formulations was evaluated using in vitro gastrointestinal digestion and enzyme inhibition assays, which provide valuable mechanistic information but cannot fully reproduce the complexity of human digestion, microbiome interactions, or inter-individual variability. Similarly, digital sensory analysis provides complementary insights into consumer perception but cannot completely predict long-term purchasing behavior or market acceptance. In addition, storage stability was evaluated over 28 days under refrigerated conditions; therefore, extended shelf-life studies under different storage conditions are required. Future investigations should include in vivo validation, longer-term stability assessment, and larger consumer studies to further establish the biological relevance, safety, and commercial potential of protein–β-glucan nanoparticle-based beverage systems.

Overall, the present findings demonstrate that microbial protein–β-glucan nanoparticles represent a promising naturally derived functional ingredient system for multifunctional functional beverages, combining improved physicochemical stability, enhanced gastrointestinal performance, increased in vitro enzyme inhibitory activity, and high consumer acceptability within a food-grade delivery system.

## 5. Conclusions

In this study, a peach juice enriched with protein–β-glucan nanoparticles was successfully developed and comprehensively characterized through an integrated evaluation of physicochemical properties, gastrointestinal digestion behavior, functional activity, and sensory performance.

Nanoparticle incorporation improved the technological and functional characteristics of the juice by enhancing colloidal stability, β-glucan bioaccessibility, antioxidant retention, and inhibitory activity against carbohydrate-hydrolyzing enzymes, including α-amylase and α-glucosidase. The high protein digestibility observed across formulations further indicates that nanoparticle assembly preserved enzymatic accessibility and did not negatively affect gastrointestinal protein utilization.

Importantly, these functional improvements were achieved without compromising consumer acceptance. Formulations V3 and V4 showed the highest overall liking and purchase intention scores, demonstrating that the incorporation of protein–β-glucan nanoparticles can enhance functional performance while maintaining desirable sensory attributes within a real food matrix.

The main contribution of this study is the development of thermally induced microbial protein–β-glucan nanoparticles as multifunctional structuring and delivery systems for fruit-based beverages. By integrating nanoscale characterization, gastrointestinal bioaccessibility assessment, enzyme inhibition analysis, digital sensory profiling, and multivariate statistical evaluation, this work provides comprehensive evidence of their technological and functional potential.

Overall, microbial protein–β-glucan nanoparticles represent a promising approach for improving the stability and functional performance of beverage systems. Future investigations should focus on in vivo validation, human intervention studies, extended storage evaluation, and larger consumer trials to further establish their physiological relevance and practical applicability.

## Figures and Tables

**Table 1:** Physicochemical properties of protein–β-glucan nanoparticles (in juice matrix, day 0).

Sample	n	Particle Size (nm)	PDI	Zeta Potential (mV)
		$\bar{x}$ ± SD	$\bar{x}$ ± SD	$\bar{x}$ ± SD
V3 (0.1% NP)	3	176.25 ± 11.52 ^a^	0.24 ± 0.12 ^a^	−19.63 ± 1.21 ^a^
V4 (0.3% NP)	3	228.74 ± 14.91 ^b^	0.29 ± 0.03 ^b^	−23.84 ± 1.48 ^b^

^a, b^—different letters within a column indicate statistically significant differences according to *t*-test (*p* < 0.05).

**Table 2:** Stability of protein–β-glucan nanoparticles in peach juice (4 °C, 28 days).

Time (Days)	n	Sample	Size (nm)	PDI	Zeta (mV)
		$\bar{x}$ ± SD	$\bar{x}$ ± SD	$\bar{x}$ ± SD	$\bar{x}$ ± SD
0	3	V3	176.25 ± 11.38 ^a^	0.24 ± 0.08 ^a^	−19.63 ± 0.17 ^a^
7	3	V3	179.84 ± 12.06 ^b^	0.25 ± 0.09 ^a^	−19.12 ± 0.09 ^a^
14	3	V3	184.57 ± 12.73 ^c^	0.26 ± 0.01 ^b^	−18.58 ± 0.31 ^b^
28	3	V3	191.36 ± 13.52 ^d^	0.27 ± 0.01 ^b^	−18.04 ± 0.18 ^b^
0	3	V4	228.74 ± 14.92 ^a^	0.29 ± 0.07 ^a^	−23.84 ± 0.36 ^a^
7	3	V4	233.68 ± 15.21 ^b^	0.30 ± 0.05 ^b^	−23.11 ± 0.28 ^a^
14	3	V4	240.46 ± 16.08 ^c^	0.31 ± 0.09 ^c^	−22.37 ± 0.45 ^b^
28	3	V4	248.93 ± 17.04 ^d^	0.33 ± 0.04 ^d^	−21.63 ± 0.52 ^c^

^a, b, c, d^—values for the same parameter and the same sample, among different days, marked with different letters, are statistically significantly different (*p* < 0.05), ANOVA followed by Tukey’s post hoc test.

**Table 3:** Viscosity and turbidity of peach juice formulations containing protein–β-glucan nanoparticles.

Sample	n	Viscosity (mPa·s)	Turbidity (NTU)
		$\bar{x}$ ± SD	$\bar{x}$ ± SD
V1	3	2.41 ± 0.08 ^a^	42.35 ± 1.21 ^a^
V2	3	2.18 ± 0.06 ^b^	28.74 ± 1.03 ^b^
V3	3	2.76 ± 0.09 ^c^	61.58 ± 1.47 ^c^
V4	3	3.12 ± 0.11 ^d^	78.92 ± 1.65 ^d^

^a, b, c, d^—different superscript letters within the same column indicate significant differences (*p* < 0.05) according to one-way ANOVA followed by Tukey’s post hoc test.

**Table 4:** β-Glucan content and bioaccessibility during in vitro gastrointestinal digestion of peach juice.

Variant	n	Initial β-Glucan (mg/100 mL)	Gastric Phase (mg/100 mL)	Gastric (% Bioaccessibility)	Intestinal Phase (mg/100 mL)	Intestinal (% Bioaccessibility)
		$\bar{x}$ ± SD	$\bar{x}$ ± SD	$\bar{x}$ ± SD	$\bar{x}$ ± SD	$\bar{x}$ ± SD
V1	3	5.35 ± 1.21 ^aA^	3.41 ± 0.21 ^aB^	63.72 ± 1.91 ^aC^	4.59 ± 0.25 ^aD^	85.92 ± 2.11 ^aE^
V2	3	12.12 ± 1.02 ^bA^	8.89 ± 0.31 ^bB^	73.40 ± 2.05 ^bC^	10.12 ± 0.38 ^bD^	83.67 ± 1.82 ^bE^
V3	3	25.88 ± 2.76 ^cA^	17.65 ± 1.45 ^cB^	68.27 ± 1.09 ^cC^	21.95 ± 1.51 ^cD^	84.86 ± 2.09 ^bE^
V4	3	40.27 ± 2.89 ^dA^	29.95 ± 1.52 ^dB^	74.45 ± 1.16 ^dC^	35.02 ± 0.63 ^dD^	87.09 ± 2.14 ^cE^

^a, b, c, d^—values for the same parameter among different variants, marked with different letters, are statistically significantly different (*p* < 0.05), ANOVA followed by Tukey’s post hoc test. ^A, B, C, D, E^—values for the same variant across different parameters, marked with different letters, are statistically significantly different (*p* < 0.05), ANOVA followed by Tukey’s post hoc test.

**Table 5:** Protein digestibility during in vitro gastrointestinal digestion of peach juice variants.

Variant	n	Gastric Phase (% Digestibility)	Intestinal Phase (% Digestibility)
		$\bar{x}$ ± SD	$\bar{x}$ ± SD
V1	3	32.45 ± 1.72 ^a^	71.32 ± 2.11 ^a^
V2	3	34.12 ± 1.89 ^b^	73.18 ± 1.24 ^b^
V3	3	30.88 ± 2.65 ^c^	69.74 ± 2.03 ^c^
V4	3	36.24 ± 1.95 ^d^	75.46 ± 2.19 ^d^

^a, b, c, d^—values for the same parameter among different variants, marked with different letters, are statistically significantly different (*p* < 0.05), ANOVA followed by Tukey’s post hoc test.

**Table 6:** Antioxidant retention during in vitro gastrointestinal digestion of peach juice variants.

Variant	n	DPPH (μmol TE/100 mL)	DPPH Retention (%)	FRAP (μmol TE/100 mL)	FRAP Retention (%)
		$\bar{x}$ ± SD	$\bar{x}$ ± SD	$\bar{x}$ ± SD	$\bar{x}$ ± SD
V1	3	14.78 ± 0.74 ^a^	79.01 ± 1.12 ^a^	12.23 ± 0.61 ^a^	79.08 ± 1.03 ^a^
V2	3	18.15 ± 0.91 ^b^	81.00 ± 1.11 ^b^	15.90 ± 0.79 ^b^	81.75 ± 1.09 ^b^
V3	3	23.45 ± 1.12 ^c^	79.87 ± 2.05 ^a^	21.35 ± 0.67 ^c^	82.68 ± 1.97 ^c^
V4	3	25.78 ± 1.28 ^d^	81.73 ± 1.22 ^b^	23.50 ± 0.18 ^d^	83.96 ± 1.15 ^d^

^a, b, c, d^—values for the same parameter among different variants, marked with different letters, are statistically significantly different (*p* < 0.05), ANOVA followed by Tukey’s post hoc test.

**Table 7:** β-Glucan bioaccessibility and release kinetics during in vitro gastrointestinal digestion of peach juice variants.

Variant	n	β-Glucan Initial (mg/100 mL)	β-Glucan Bioaccessibility (%)	Release Rate Constant k (min^−1^)	R^2^	AUC (mg·min/100 mL)
		$\bar{x}$ ± SD	$\bar{x}$ ± SD	$\bar{x}$ ± SD	$\bar{x}$ ± SD	$\bar{x}$ ± SD
V1	3	5.35 ± 1.21 ^a^	85.89 ± 0.11 ^a^	0.022 ± 0.002 ^a^	0.942 ± 0.011 ^a^	43.97 ± 1.87 ^a^
V2	3	12.12 ± 1.02 ^b^	83.66 ± 1.88 ^b^	0.024 ± 0.003 ^b^	0.951 ± 0.009 ^a^	99.87 ± 2.51 ^b^
V3	3	25.88 ± 2.76 ^c^	84.83 ± 2.04 ^c^	0.021 ± 0.002 ^a^	0.963 ± 0.008 ^a^	209.95 ± 3.04 ^c^
V4	3	40.27 ± 2.89 ^d^	86.97 ± 1.14 ^a^	0.027 ± 0.001 ^c^	0.971 ± 0.006 ^b^	349.87 ± 3.11 ^d^

^a, b, c, d^—values for the same parameter among different variants, marked with different letters, are statistically significantly different (*p* < 0.05), ANOVA followed by Tukey’s post hoc test.

**Table 8:** In vitro α-Amylase inhibitory activity of peach juice formulations.

Variant	n	Inhibition (%)	IC_50_ (mg/mL)
		$\bar{x}$ ± SD	$\bar{x}$ ± SD
V1	3	32.47 ± 1.13 ^a^	72.34 ± 1.21 ^a^
V2	3	38.12 ± 0.87 ^b^	64.58 ± 1.76 ^b^
V3	3	41.29 ± 1.04 ^c^	59.42 ± 0.89 ^c^
V4	3	45.36 ± 1.21 ^d^	53.87 ± 1.02 ^d^

^a, b, c, d^—values for the same parameter among different variants, marked with different letters, are statistically significantly different (*p* < 0.05), ANOVA followed by Tukey’s post hoc test.

**Table 9:** In vitro α-Glucosidase inhibitory activity and synergy index (SI) of peach juice formulations.

Variant	n	Inhibition (%)	IC_50_ (mg/mL)	Synergy Index (SI)
		$\bar{x}$ ± SD	$\bar{x}$ ± SD	$\bar{x}$ ± SD
V1	3	28.63 ± 1.94 ^a^	79.25 ± 1.56 ^a^	0.98 ± 1.04 ^a^
V2	3	34.57 ± 2.06 ^b^	69.12 ± 1.88 ^b^	1.02 ± 1.11 ^b^
V3	3	39.21 ± 1.17 ^c^	61.47 ± 1.74 ^c^	1.08 ± 2.37 ^b^
V4	3	43.48 ± 1.34 ^d^	56.39 ± 1.92 ^d^	1.15 ± 1.07 ^c^

^a, b, c, d^—values for the same parameter among different variants, marked with different letters, are statistically significantly different (*p* < 0.05), ANOVA followed by Tukey’s post hoc test.

**Table 10:** Consumer acceptance of peach juice formulations (hedonic scores).

Sensory Attribute	V1	V2	V3	V4
	$\bar{x}$ ± SD	$\bar{x}$ ± SD	$\bar{x}$ ± SD	$\bar{x}$ ± SD
Appearance liking	6.12 ± 0.21 ^a^	6.45 ± 0.38 ^b^	6.63 ± 0.31 ^c^	7.82 ± 0.14 ^d^
Taste liking	5.98 ± 0.43 ^a^	6.27 ± 0.20 ^b^	7.52 ± 1.07 ^c^	6.74 ± 0.35 ^d^
Mouthfeel liking	5.87 ± 0.39 ^a^	6.21 ± 0.61 ^b^	6.48 ± 0.26 ^c^	7.69 ± 0.32 ^d^
Overall liking	5.94 ± 0.42 ^a^	6.28 ± 0.39 ^b^	8.53 ± 0.51 ^c^	8.77 ± 0.24 ^c^
Purchase intent (% willing to buy)	62.32 ± 1.37 ^a^	68.57 ± 1.91 ^b^	73.11 ± 1.15 ^c^	78.59 ± 1.69 ^d^

^a, b, c, d^—values for the same parameter among different variants, marked with different letters, are statistically significantly different (*p* < 0.05), ANOVA followed by Tukey’s post hoc test; n = 60 participants.

**Table 11:** Digital sensory analysis of peach juice formulations.

Type	Parameter	V1	V2	V3	V4
		$\bar{x}$ ± SD	$\bar{x}$ ± SD	$\bar{x}$ ± SD	$\bar{x}$ ± SD
**Facial Expression**	Lip Press	0.42 ± 0.16 ^a^	0.39 ± 0.05 ^b^	0.37 ± 0.05 ^c^	0.34 ± 0.04 ^d^
	Lip Suck	0.31 ± 0.14 ^a^	0.29 ± 0.03 ^b^	0.28 ± 0.04 ^b^	0.26 ± 0.03 ^c^
	Mouth Open	0.47 ± 0.05 ^a^	0.50 ± 0.06 ^b^	0.52 ± 0.05 ^c^	0.54 ± 0.05 ^d^
**Head Orientation**	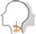 Yaw	2.12 ± 0.29 ^a^	2.05 ± 0.18 ^b^	2.01 ± 0.07 ^c^	1.97 ± 0.16 ^d^
	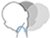 Roll	1.08 ± 0.22 ^a^	1.05 ± 0.01 ^b^	1.02 ± 0.62 ^b^	0.99 ± 0.11 ^c^
**Emotion**	Surprise	0.18 ± 0.03 ^a^	0.20 ± 0.03 ^b^	0.22 ± 0.04 ^c^	0.23 ± 0.03 ^c^
	Joy	0.62 ± 0.25 ^a^	0.64 ± 0.04 ^b^	0.67 ± 0.05 ^c^	0.70 ± 0.04 ^d^
	Disgust	0.12 ± 0.02 ^a^	0.11 ± 0.02 ^a^	0.10 ± 0.02 ^a^	0.09 ± 0.01 ^b^
	Relaxed	0.53 ± 0.04 ^a^	0.55 ± 0.05 ^b^	0.57 ± 0.05 ^c^	0.60 ± 0.14 ^d^
**Emoji**	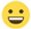 Smiley	0.58 ± 0.05 ^a^	0.61 ± 0.04 ^b^	0.64 ± 0.07 ^c^	0.67 ± 0.04 ^d^
	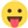 Stuck Out Tongue	0.21 ± 0.03 ^a^	0.19 ± 0.03 ^b^	0.18 ± 0.03 ^b^	0.22 ± 0.02 ^c^
	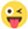 Stuck Out Tongue Winking Eye	0.15 ± 0.02 ^a^	0.14 ± 0.02 ^a^	0.13 ± 0.02 ^a^	0.14 ± 0.01 ^a^
	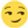 Smirk	0.32 ± 0.04 ^a^	0.30 ± 0.04 ^b^	0.29 ± 0.03 ^b^	0.27 ± 0.03 ^c^

^a, b, c, d^—values for the same parameter among different variants, marked with different letters, are statistically significantly different (*p* < 0.05), ANOVA followed by Tukey’s post hoc test; n = 60 participants.

**Table 12:** Exploratory multivariate analysis inputs and observed outcomes for peach juice variants.

Variant	β-Glucan (mg/100 mL)	Protein (mg/100 mL)	Release Rate k (min^−1^)	Storage (Days)	Predicted α-Amylase IC_50_ (mg/mL)	Predicted α-Glucosidase IC_50_ (mg/mL)
V1	5.35 ± 1.42 ^a^	0.41 ± 0.03 ^a^	0.023	0	72.32 ± 2.17 ^a^	79.25 ± 2.09 ^a^
V1	4.98 ± 1.36 ^a^	0.41 ± 0.02 ^a^	0.023	28	71.57 ± 2.21 ^a^	78.51 ± 2.03 ^a^
V2	12.12 ± 3.57 ^b^	0.52 ± 0.03 ^b^	0.025	0	64.65 ± 1.14 ^b^	69.14 ± 3.06 ^b^
V2	11.92 ± 1.61 ^b^	0.51 ± 0.08 ^b^	0.025	28	63.80 ± 3.08 ^b^	68.43 ± 2.11 ^b^
V3	25.88 ± 1.02 ^c^	1.20 ± 0.05 ^c^	0.022	0	59.41 ± 1.03 ^c^	61.58 ± 3.23 ^c^
V3	24.87 ± 3.08 ^c^	1.19 ± 0.04 ^c^	0.022	28	57.82 ± 1.10 ^c^	60.10 ± 2.16 ^c^
V4	40.27 ± 2.23 ^d^	1.81 ± 0.07 ^d^	0.027	0	53.91 ± 3.27 ^d^	56.49 ± 3.26 ^d^
V4	40.12 ± 3.18 ^d^	1.81 ± 0.06 ^d^	0.027	28	52.56 ± 3.34 ^d^	55.07 ± 2.02 ^d^

^a–d^—values for the same parameter and the same storage day, among different variants, marked with different letters, are statistically significantly different (*p* < 0.05), ANOVA followed by Tukey’s post hoc test. Model performance: α-Amylase IC50 model: R^2^ = 0.912; α-Glucosidase IC50 model: R^2^ = 0.894. The regression-based predictive models demonstrated good agreement between predicted and observed values.

## Data Availability

The original contributions presented in this study are included in the article. Further inquiries can be directed to the corresponding authors.
